# Association between sleep duration and cardiac structure in youths at risk for metabolic syndrome

**DOI:** 10.1038/srep39017

**Published:** 2016-12-14

**Authors:** Dan Feng, Jihui Zhang, Junling Fu, Heng Wu, Yonghui Wang, Lujiao Li, Yanglu Zhao, Ming Li, Shan Gao

**Affiliations:** 1 Department of Endocrinology, Beijing Chaoyang Hospital, Capital Medical University, Beijing, China; 2 Department of Psychiatry, Faculty of Medicine, The Chinese University of Hong Kong, Hong Kong SAR, China; 3 Department of Endocrinology, Key Laboratory of Endocrinology, National Health and Family Planning Commission, Peking Union Medical College Hospital, Chinese Academy of Medical Sciences and Peking Union Medical College, Beijing, China; 4 Epidemiology Department, Fielding School of Public Health, University of California Los Angeles, USA

## Abstract

The evidence for a link between sleep duration and cardiovascular risk is accumulating in youths, but no study has yet investigated the relationship between sleep duration and change of cardiac structure. In this study, we recruited 559 youths aged 14–28 years from the cohort of Beijing Child and Adolescent Metabolic Syndrome Study. Questionnaire, color Doppler echocardiography, oral glucose tolerance test and blood biomarkers analyses were performed. We found that sleep duration was negatively correlated with body mass index, waist circumstance, and HbA1c (all *P* < 0.05), but not with adiponectin and leptin. Meanwhile, participants with shorter sleep duration (≤7 h) had larger interventricular septal diastolic thickness, left ventricular (LV) end-diastolic diameter, LV posterior wall thickness, LV mass (LVM), and LV mass index (LVMI), compared to participants in 7–9 h/night or >9 h/night group. Findings remained significant after adjustment for the major confounding factors (*P* < 0.05). Multivariate regression modeling revealed that each additional hour of sleep was associated with smaller LVM (β: −3.483, *P* < 0.0001) and LVMI (β: −0.815, *P* < 0.0001). Our findings suggest that short sleep has a possible direct effect on cardiac remodeling, occurring already at young ages.

Chronic sleep restriction has become a pervasive problem in modern society^1^,^2^. Sleep is not only a fundamental biological phenomenon, but also plays an important role in the promotion of growth, development, and maturity in adolescence. Short sleep duration has been linked to a number of cardio-metabolic diseases, such as obesity, insulin resistance, type 2 diabetes mellitus, hypertension, and metabolic syndrome in both children and adults^3^,^4^,^5^,^6^,^7^. Furthermore, prospective studies have shown that sleep duration predicts incident cardiovascular diseases (CVD)^8^. The relation between short sleep duration and cardiovascular disease incidence could be partly attributed to an effect of short sleep on the above-mentioned traditional cardiovascular disease risk factors.

Nevertheless, recent studies have shown several novel CVD risk factors, such as adipokine leptin^9^, adiponectin^10^, and inflammatory marker high-sensitivity C-reactive protein (hs-CRP)^11^, being linked to short sleep duration. On the other hand, these cardio-metabolic factors have also been found to contribute to cardiac abnormalities, even in the youth, with early changes of cardiac geometry and function^12^,^13^,^14^. Therefore, these findings suggest that short sleep may lead to cardiac abnormalities through impairing cardio-metabolic functions, which in turn mediates in the effects of sleep on cardiovascular diseases. However, to our knowledge, no study directly investigated the association of cardiac structure and function changes with sleep duration. Since echocardiography has been shown to be a sensitive tool for detecting the functions and abnormalities of cardiac structure^15^,^16^, in this study, on the basis of a cohort study of the Beijing Child and Adolescent Metabolic Syndrome (BCAMS), we aimed to determine the associations of sleep duration with parameters of cardiac structure as measured by color Doppler echocardiography. In addition, we also examined whether the cardio-metabolic risk factors, which are known to be associated with sleep duration in adults, could play a role in mediating in these associations during young ages.

## Methods

### Participants

Subjects were participants in the cohort of BCAMS^17^,^18^. The BCAMS is a prospective cohort study, which aims to identify cardiovascular risk factors from childhood to adulthood. The baseline population-based survey was conducted in a representative sample (n = 19,593, 50% boys) of school children in Beijing aged 6–18 years in 2004. Totally, about 4500 participants were identified as being at a high risk of cardiovascular disease due to having one of the following abnormities: overweight as defined by body mass index (BMI) between 85^th^ percentiles and 95^th^ percentiles for age and sex, high blood pressure (≥90^th^ percentiles for age and sex), elevated lipids (total cholesterol ≥5.2 mmol/L, triglyceride ≥1.7 mmol/L), and/or fasting blood glucose (≥5.6 mmol/L) based on finger capillary blood tests. The current study was the follow-up study beginning in 2013. Participants were recruited consecutively through various modalities (phone, text, and/or email) and underwent medical examination at a center in the Beijing Chaoyang Hospital. All of the experimental protocols for the follow-up study were approved by the Ethics Committee at the Beijing Chaoyang Hospital, and all the methods were performed in accordance with relevant guidelines and regulations. Participants aged 18 years old or above gave written informed consent. For the participants aged under 18 years old, their parent(s) or guardian(s) gave written consent and all the participants gave written assent to take part in the study. A total of 564 subjects were enrolled, however, 5 of them refused to undergo echocardiogram. Thus a total of 559 participants had complete data and were included into this analysis

### Clinical measurements

After a minimum of 10 hour overnight fasting, participants came to the hospital medical center for an in-person study visit. Height, weight, waist circumference, and fat percentage (FAT%) were measured by trained field workers. Participants removed bulky clothing and shoes prior to measurements. Height was measured to the nearest 0.1 cm using a portable stadiometer. Waist circumference was measured midway between the lowest rib and the top of the iliac crest. FAT% was measured to the nearest 0.1 kg using a TANITA Body Composition Analyzer (Model TBF-300A, Tanita, Japan). Measurements of right arm systolic and diastolic blood pressure (SBP and DBP) were performed 3 times, each with 10 minutes apart. The mean values of the latter two measurements were used. BMI was calculated as weight divided by height squared. Life style factors and health history information were obtained by standardized questionnaires. Cigarette smoking was defined as current, former, or never.

### Laboratory measurements

An oral glucose tolerance test (OGTT) using 75 g glucose load was performed on each subject in the morning after 10-hour fasting. Blood glucose levels in fasting, 0.5-hour and 2-hour were measured by hexokinase method. HbA1c was analyzed using the TOSOH G7 automatic analysis system with high performance liquid chromatography. Blood triglycerides, total cholesterol, low-density lipoprotein cholesterol (LDL-C) and high-density lipoprotein cholesterol (HDL-C) were assayed using a standard enzymatic method. Hs-CRP was measured by immunoturbidimetric assay. Insulin, leptin and adiponectin concentrations were measured by monoclonal antibody-based sandwich enzyme-linked immunosorbent assay developed in the Key Laboratory of Endocrinology, Peking Union Medical College Hospital^19^,^20^,^21^. The intra-assay coefficient of variation (CVs) for insulin, leptin and adiponectin were <4.1%, <7.4% and <5.4%, respectively. The inter-assay CVs were <7.0%, <9.3% and <8.5%, respectively.

### Definitions

Type 2 diabetes mellitus was defined as fasting blood glucose ≥7.0 mmol/L or OGTT 2-hour blood glucose ≥11.1 mmol/L or HbA1c ≥ 6.5%^22^. Participants younger than 18 years were classified as overweight, if BMI was between the 85^th^ and 95^th^ percentile, or obese if BMI was above 95^th^ percentile^23^. Participants older than 18 years old were classified overweight if BMI ≥ 24 kg/m^2^, or obese if BMI ≥ 28 kg/m^2^. Hypertension was classified as SBP or DBP ≥ 95^th^ percentile for age, sex, and height (<18 years old) or ≥140/90 mmHg^24^. Insulin resistance was assessed by the homeostasis model assessment (HOMA-IR), calculated as fasting insulin (μIU/ mL)× fasting blood glucose (mmol/L)/22.5^25^. Metabolic syndrome was diagnosed according to the consensus definition by the International Diabetes Federation^14^.

### Sleep duration determination

A self-reported questionnaire was employed to measure sleep habits (including bed time and wake up time), sleep duration, and sleep quality. Sleep duration was asked by time bar reaching half-hourly from 5 to 13 hours per night. Since there is no evidence to prove that how long is the appropriate sleep time of adolescent in China, we grouped sleep duration into three categories: short sleepers (≤7 h/night), normal sleepers (>7 to ≤9 h/night), and long sleepers (>9 h/night). We also collected their sleep quality by a modified questionnaire including 11 questions (sleep onset delay, snoring, mouth breathing, awaking from sleep not breathing or gasping or choking, sleep apnea, sleepwalking/nightmares, body twitching, excessive sweating, enuresis, difficulty getting up, and headaches in the morning). The scores are summed as sleep quality score, with higher scores denoting poorer sleep quality.

### Echocardiography

Non-invasive transthoracic echocardiogram was performed with a LOGIQ P5 B-mode ultrasonogram equipped (LOGIQ P5, GE Ultrasound, Korea) with a 2.5–3.5 MHz probe. All images were obtained in the left decubitus position of participants to acquire parasternal long and short axis and apical four chamber views. Three cardiac measurements were measured by a sonographer who was blinded to group, including interventricular septal diastolic thickness (IVSDT), left ventricular end-diastolic diameter (LVEDD), and left ventricular posterior wall thickness (LVPWT). Cardiac structure and geometry-LV mass was calculated using the formula: LV mass (LVM) = 0.8 * {1.04 * [(LVEDD + LVPWT + IVSDT)^3^ − (LVEDD)^3^]} + 0.6; LV mass index (LVMI) (a measure of hypertrophy) was calculated by dividing LVM by height in meters raised to 2.7 (LVM/height^2.7^) to minimize the effects of age, sex^26^,^27^. Due to lack of threshold level of LVMI for Chinese youths, left ventricular hypertrophy (LVH) was defined as having an LVMI greater the sex-specific 90^th^ percentile in this population.

### Data analysis

Analyses were performed using the Statistical Package for Social Sciences (SPSS 19.0 for Windows, SPSS Inc., USA). Continuous variables were tested for normality using the Kolmogorov-Smirnov test. Non-normal distribution values such as insulin, leptin, adiponectin, and HOMA-IR were natural log-transformed to comply with normality. Comparison between the groups was achieved using the t-test or analysis of variance for continuous variables, while categorical variables were explored using the chi-square test. Linear and quadratic terms were tested in General Linear Models by using polynomial contrasts to test the linear or quadratic trend in the association between sleep duration and echocardiography parameters. Multivariate linear regression models were used to identify the independent associations of sleep duration with echocardiographic parameters including LVM and LVMI. Model 1 was adjusted for age, sex, height and weight; model 2 was adjusted for factors in model 1 plus triglyceride, HDL-C, blood pressures, HbA1c%, smoking, and physical activity and sleep quality; model 3 was adjusted further for hs-CRP, HOMA-IR, adiponectin and leptin. A *P*-value < 0.05 (two sided) was considered to be statistically significant.

## Results

### The general characteristics of the study population

The clinical, biochemical and echocardiographic features of the participants stratified by gender are shown in Table 1. The average age of all participants was 20.2 ± 2.9 years (14–28 years). Of the total 559 participants, 294 (52.6%) were males. The average sleep duration was 8.2 ± 1.3 hours. Since the cohort was designed to study metabolic syndrome, the prevalence rates of obesity and metabolic syndrome were as high as 32% and 10.4%, respectively. HDL-C, leptin, and adiponectin were higher in females when compared with males (*P* < 0.001), while triglycerides, blood pressure, smoking, moderate to high activity and all the five echocardiographic parameters were higher in males when compared with females (*P* < 0.001).

### Association of sleep duration with cardio-metabolic parameters

Demographic and cardio-metabolic characteristics by sleep duration are presented in Table 2. After adjustment for age and sex, participants with short sleep (≤7 h/night) had significantly higher BMI, FAT%, and waist circumference when compared with other groups (*P* < 0.05). No differences were found in fasting blood glucose, 2-hour blood glucose, HOMA-IR, SBP, triglycerides, total cholesterol, HDL-C, leptin, adiponectin, smoking and sleep quality among different sleep duration groups (*P* > 0.05). We found an increasing trend in the heart rate of short sleepers (≤7 h/night) but without statistical significance. However, after excluding participants with type 2 diabetes mellitus, HbA1c were significant higher in short duration group (*P* = 0.001) when compare with other two groups.

### Association of sleep duration with cardiac-parameters

**Figure 1:**
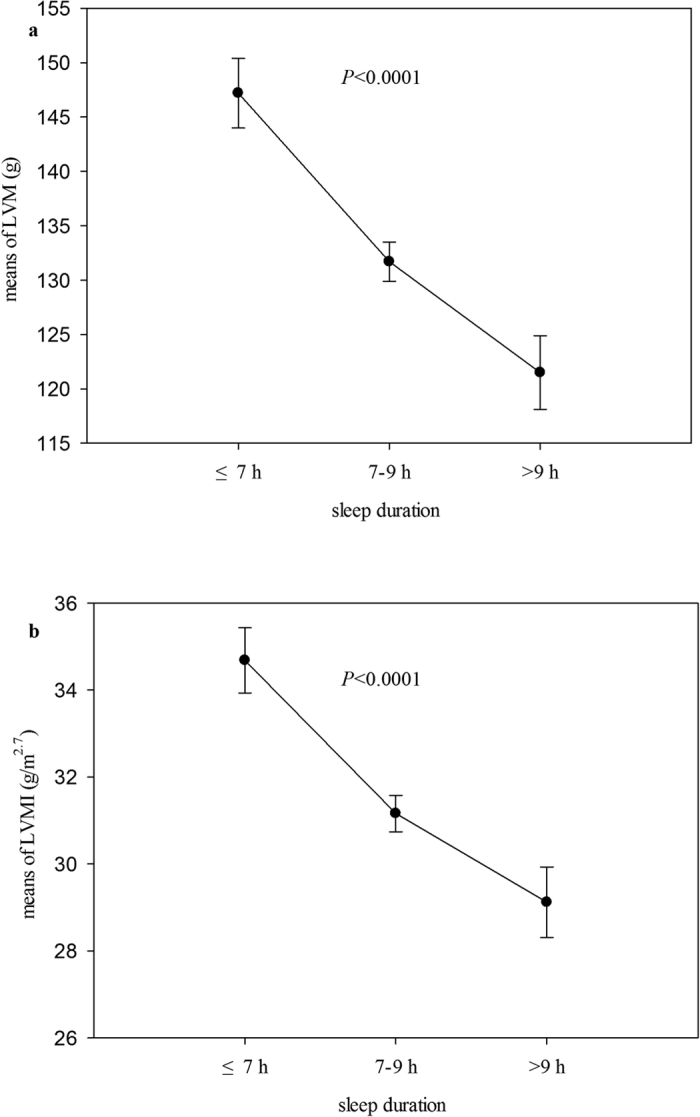
LVM **(a)** and LVMI (**b**) by sleep time. Values displayed as geometric mean concentrations and 95% confidence interval. Models adjusted for age, gender, height, weight, waist circumference, blood pressures, lipids, HbA1c, HOMA-IR, leptin, adiponectin, hs-CRP, smoking, physical activity and sleep quality.

Measures of cardiac structure stratified by groups are presented in Table 3. The study shows a significant inverse relationship between sleep duration and all the five cardiac parameters. After adjusting for age and sex, short sleepers (≤7 h/night) had larger IVSDT, LVEDD and LVPWT than others in 7–9 h/night and >9 h/night groups (*P* < 0.01). Participants in long sleep group (>9 h/night) had smaller LVEDD than those in 7–9 h/night group (*P* < 0.01). As for LVM and LVMI, the results showed more obvious trend than those in IVSDT, LVEDD and LVPWT among groups (*P* < 0.0001). Furthermore, after full adjustment for major risk factors including age, gender, height, weight, waist circumference, blood pressures, lipids, HbA1c, HOMA-IR, leptin, adiponectin and hs-CRP as well as smoking, physical activity and sleep quality, these differences in cardiac parameters among groups were not materially modified, especially in LVM and LVMI (*P* < 0.0001) (Fig. 1). In addition, the short sleep group had higher percentage of LVH than that in normal (7–9 h/night) and long sleep (>9 h/night) groups (18.3% vs. 8% vs. 6.7%, *P* < 0.05). These results confirmed that insufficient sleep (≤7 h/night) was an independent risk factor of cardiac structural changes.

When assessed as a continuous variable in linear regression models (Table 4), sleep duration was also significantly negatively associated with cardiac parameters, especially for LVM and LVMI. Fully adjusting for potentially confounding or mediating variables including age, sex, height, weight, blood pressures, lipids, HbA1c, HOMA-IR, smoking, physical activity, hs-CRP, adipokines, and sleep quality do not appreciably attenuate the associations between sleep duration and cardiac parameters. Each hour decrease in sleep duration is accompanied by 3.48 g increase in LVM and 0.82 g/m^2.7^ increase in LVMI.

## Discussion

In this cross-sectional study of adolescents at risk for metabolic syndrome, our major finding is that short sleep duration was associated with cardiac structural remodeling, such as increased IVSDT, LVEDD and LVPWT and the calculated parameters of LVM and LVMI. These associations were not likely to be confounded by a series of potential factors, including age, sex, height, weight, blood pressure, lipids, HbA1c, HOMA-IR, hs-CRP, leptin and adiponectin as well as smoking, physical activity, and sleep quality. In addition, as expected, this study also replicated well-known negative associations between sleep duration and BMI, FAT%, and waist circumference. According to our knowledge, this is the first study to explore the relationship between sleep duration and cardiac structure in youth.

Changes of cardiac structure have been shown to have a predictive role in cardiovascular outcomes^28^,^29^,^30^. LVM and LVH are markers of left ventricle remodeling, recognized as important measures to assess clinical prognosis^29^,^31^. Heart size scales are related to body size, sex, and age. As such, LVMI is calculated to minimize the effects of age, sex, and increased LVMI could independently predict cardiovascular events^30^. Established risk factors for increasing LVM and LVMI are obesity, high blood pressure, hyperglycemia, insulin resistance, systemic inflammation and adipokines abnormalities^12^,^32^,^33^,^34^. In this study, there was a significant increase in LVM and LVMI with decreased sleep duration, suggesting that insufficient sleep was also an independent risk factor of cardiac structural changes. Meanwhile, the commonly known risk factor for LVH such as BMI, and waist circumference were also found to be associated with sleep duration in current study. Therefore, to explore whether these potential confounding factors could bias the association of sleep duration with LVM and LVMI, we adjusted for age, sex, height, weight, waist circumference, blood pressures, HbA1c, lipids, and current life style factors, and even the novel biomarkers for CVD including hs-CRP and adipokine leptin and adiponectin^35^. However, our findings remained significant after full adjustment, which indicated that sleep time independently predicted LVM and LVMI. In addition, although lack of threshold level of LVMI for Chinese youths, the estimated percentage of LVH, as defined by LVMI> the sex-specific 90^th^ percentile in this population, was also higher in short sleep group than in longer sleep groups. In this regard, our findings indicated short sleep duration may serve as an additional novel risk factor of LVH. In view of the close association of sleep duration with cardiovascular diseases^8^,^9^, our findings suggest that the cardiac remodeling may be the potential pathway underlying short sleep duration and cardiovascular diseases.

The mechanisms that underlie these associations between short sleep duration and cardiac remodeling are unclear. To explore whether the above-mentioned conventional CVD risk factors and novel potential factors acted as mediators in the relationship between sleep duration and cardiac structure, we added these variables to the multivariate models to see whether they resulted in attenuation in the relationship. Since there was no significant attenuation, this is consistent with these variables not serving as mediating variables. Therefore, some additional mediating factors warrant to be considered, such as sympathetic tone, other peptides, transmitters, and their receptors controlling cardiovascular function. For instance, sleep has a beneficial effect on the sympathetic nervous system^36^. In clinical study, Zhang *et al*. found that insufficient sleepers had a higher 24-h urinary norepinephrine and epinephrine levels^37^, which serve as a reliable indicator of overall sympathetic activity. In line with this, experimental study has shown continuous intravenous infusion of norepinephrine induced hypertrophy of the left ventricle in rats^38^. Thus, sympathetic nervous system activity should be considered in further mechanism study. We analysed the resting heart rate, while there was no difference among three groups. We found no difference in the resting heart rate between the 3 sleep duration groups, however, we did find an increasing trend in the heart rate among those reporting short sleep durations (≤7 h/night) but this trend was not statistically significant. Other indicators of sympathetic drive that could influence resting heart rate therefore represent opportunities for future research. In addition, besides C-reactive protein, adiponectin and leptin, more functionally prominent inflammation markers and adipokines such as IL-6, TNF-alpha, resistin, and retinol binding protein 4 should be perused in further mechanism investigation^35^,^39^.

In the present study, we also explored the associations between sleep duration and several well–known cardio-metabolic risk factors. We replicated the finding that short sleep duration is negatively correlated with obesity-related anthropometric measurements^3^, however, we did not find significant relationships between sleep duration and other metabolic parameters such as HOMA-IR, blood pressure, lipids, leptin, or adiponectin. Those parameters were previously demonstrated to be associated with sleep duration in some^3^,^4^,^5^,^6^,^7^,^8^,^9^,^10^ but not all^8^,^40^ studies. These discrepancies could be explained by differences in samples, sleep duration categories, and varied control for confounders. However, replication of some expected associations between sleep duration and the study outcomes in this study also provides validity in terms of the methodology used here.

Strengths of this study include a relatively large cohort, the standardized protocol with detailed and well-characterized cardio-metabolic phenotype and the novel finding that short sleep duration was associated with cardiac structural changes. Despite the strength, there are some key limitations to our study. First, the sample in the present study was drawn from youths at risk of cardiovascular diseases; future replication study is needed to evaluate the generalizability of our findings to older adults and other ethnical populations. Secondly, information about sleep duration and quality were self-reported by participants. Although questionnaire has been commonly used in epidemiological studies^2^,^3^,^4^,^5^,^6^,^7^,^8^,^9^, and a good correlation between objectively measured and self-reported sleep duration has been observed^41^,^42^, the subjective measure still may compromise the accuracy compared with the gold-standard polysomnography. Thus, our findings need to be validated by objective measures in future studies. Lastly, we found a relationship between short sleep duration and abnormal cardiac geometry, but causality is difficult to infer. Our ongoing follow-up observation and/or experimental studies are required to elucidate the causal relationship between sleep duration and cardiac abnormality.

## Conclusions

In conclusion, our finding that short sleep duration is associated with the adverse changes of cardiac structure in youth highlights the importance of getting adequate sleep to protect cardiac structure. Future work should focus on applying longitudinal and experimental designs to determine causality.

## Additional Information

**How to cite this article**: Feng, D. *et al*. Association between sleep duration and cardiac structure in youths at risk for metabolic syndrome. *Sci. Rep.* **6**, 39017; doi: 10.1038/srep39017 (2016).

**Publisher's note:** Springer Nature remains neutral with regard to jurisdictional claims in published maps and institutional affiliations.

## Figures and Tables

**Table 1:** The characteristics of the study population.

	Total	Male	Female	P value
n	559	294	265	—
Age, year	20.2 (2.9)	20.0 (3.0)	20.4 (2.8)	0.11
sleep hours, hour	8.20 (1.25)	8.17 (1.17)	8.24 (1.34)	0.501
sleep quality score	2.2 (1.8)	2.3 (1.9)	2.1 (1.8)	0.196
Moderate to high activity, h/week	2.2 (1.9)	3.2 (3.8)	1.7 (2.0)	<0.001
Smoke, n (%)	109 (19.5%)	75 (25.5%)	34 (12.8%)	<0.001
BMI, kg/m^2^	25.7 (5.7)	27.0 (5.8)	24.3 (5.3)	<0.001
WC, cm	85.2 (14.6)	90.5 (14.6)	79.3 (12.1)	<0.001
FAT%	30.43 (10.26)	27.92 (9.38)	33.21 (10.49)	<0.001
Fasting blood glucose, mmol/L	4.92 (0.69)	5.00(0.86)	4.82 (0.41)	0.001
2 h-glucose, mmol/L	6.06 (1.84)	6.17 (2.07)	5.95 (1.54)	0.179
HbA1c, %	5.38 (0.48)	5.42 (0.54)	5.33 (0.40)	0.038
Fasting insulin, mIU/L	9.1 (7.9)	9.5 (8.2)	8.7 (7.6)	0.236
2 h-insulin, mIU/L	51.7 (59.3)	49.9 (51.1)	53.6 (67.2)	0.468
HOMA-IR	2.04 (1.92)	2.15 (2.01)	1.92 (1.81)	0.146
SBP, mmHg	115 (14)	121 (14)	108 (11)	<0.001
DBP, mmHg	73 (10)	76 (10)	70 (10)	<0.001
Triglycerides, mmol/L	1.13 (0.83)	1.25 (1.01)	1.01 (0.54)	0.001
Total cholesterol, mmol/L	4.35 (0.92)	4.29 (0.86)	4.41 (0.99)	0.131
LDL-C, mmol/L	2.53 (0.79)	2.56 (0.72)	2.50 (0.86)	0.371
HDL-C, mmol/L	1.44 (0.32)	1.34 (0.28)	1.54 (0.34)	<0.001
Hs-CRP, mg/L	1.81 (2.54)	1.73 (2.30)	1.90 (2.77)	0.42
Leptin, ng/mL	2.87 (2.80)	1.85 (1.96)	3.99 (3.15)	<0.001
Adiponectin, ug/mL	8.09 (5.41)	7.04 (4.12)	9.27 (6.36)	<0.001
Overweight, n (%)	135 (24%)	75 (26%)	60 (23%)	<0.001
Obesity, n (%)	181 (32%)	121 (41%)	60 (23%)	<0.001
T2DM, n (%)	9 (2%)	6 (2%)	3 (1%)	0.218
Hypertension, n (%)	53 (9%)	45 (15%)	8 (3%)	<0.001
MS, n (%)	58 (10.4%)	46 (15.6%)	12 (4.5%)	<0.001
Resting heart rate, bpm	79.2(17.6)	77.0 (12.8)	81.5(11.9)	<0.001
IVSDT, cm	0.89 (0.12)	0.93 (0.12)	0.85 (0.10)	<0.001
LVEDD, cm	4.42 (0.49)	4.65(0.45)	4.17 (0.40)	<0.001
LVPWT, cm	0.89 (0.11)	0.94 (0.10)	0.84 (0.10)	<0.001
LVM, g	133.1 (39.1)	153.4 (36.9)	111.7 (28.4)	<0.001
LVMI, g/m^2.7^	31.51 (8.00)	33.23 (8.32)	29.71 (7.23)	<0.001

Data are the means (SD) or N (%). Abbreviations: BMI = body mass index; WC = waist circumference; FAT% = body fat percentage; HOMA-IR = insulin resistance index; SBP = systolic blood pressure; DBP =  diastolic blood pressure; LDL-C = low-density lipoprotein cholesterol; HDL-C = high-density lipoprotein cholesterol; Hs-CRP = high-sensitivity c-reactive protein; IVSDT = inter ventricular septal diastolic thickness; LVEDD = left ventricular end-diastolic diameter; LVPWT = left ventricular posterior wall thickness; LVM = left ventricular mass; LVMI = left ventricular mass index; T2DM = type 2 diabetes mellitus; MS = metabolic syndrome.

**Table 2:** Clinical features stratified by sleep duration.

	Sleep hours			P value
	≤7 h	7–9 h	>9 h	
n	114	349	96	—
sleep quality score	2.1(1.7)	2.1(1.8)	2.3(2.0)	0.715
Moderate to high activity, h/week	3.0(4.6)	2.5(2.8)	2.0(2.8)^*^	0.055
Smoke, n (%)	23(20.2)	68(19.5)	18(18.8)	0.967
Resting heart rate,bmp	80.4(1.2)	78.6(0.7)	79.8(1.3)	0.346
BMI, kg/m^2^	27.4 (0.52)	25.3 (0.30)**	25.2 (0.56)**	0.001
FAT%	33.0 (0.9)	29.8 (0.5)^**^	29.5 (1.0)^*^	**0.008**
WC, cm	88.3 (1.3)	84.7 (0.7)^*^	83.6 (1.4)^*^	**0.021**
SBP, mmHg	116 (1)	114 (1)	116 (1)	0.208
DBP, mmHg	75 (1)	72 (1)^*^	75 (1)^†^	**0.027**
Fasting blood glucose, mmol/L	4.88 (0.07)	4.93 (0.04)	4.93 (0.07)	0.758
2h-glucose, mmol/L	6.09 (0.18)	6.04 (0.10)	6.11 (0.20)	0.946
HbA1c, %	5.39 (0.03)	5.35 (0.02)	5.24 (0.03)^**^	**0.001**
Ln fasting insulin, mIU/L	2.02 (0.07)	1.93 (0.04)	1.88 (0.08)	0.384
Ln 2h-insulin, mIU/L	3.69 (0.08)	3.58 (0.04)	3.60 (0.08)	0.451
Ln HOMA-IR	0.48 (0.07)	0.40 (0.04)	0.36 (0.08)	0.470
Triglycerides, mmol/L	1.21 (0.08)	1.09 (0.04)	1.19 (0.08)	0.316
Total cholesterol, mmol/L	4.38 (0.09)	4.30 (0.05)	4.49 (0.09)	0.152
LDL-C, mmol/L	2.58 (0.07)	2.48 (0.04)	2.67 (0.08)^†^	0.087
HDL-C, mmol/L	1.41 (0.03)	1.45 (0.02)	1.42 (0.03)	0.558
Ln hs-CRP, mg/L	0.43 (0.12)	0.006 (0.07)**	0.183 (0.13)*	0.050
Ln leptin, ng/mL	0.66(0.09)	0.58 (0.05)	0.52 (0.09)	0.496
Ln adiponectin, ug/mL	1.81 (0.06)	1.91 (0.03)	1.97 (0.07)	0.160

Data are the means (SE) adjusted for age and sex. Abbreviations: BMI = body mass index; WC = waist circumference; FAT% = body fat percentage; HOMA-IR = insulin resistance index; SBP = systolic blood pressure; DBP = diastolic blood pressure; LDL-C = low-density lipoprotein cholesterol; HDL-C = high-density lipoprotein cholesterol; Hs-CRP = high-sensitivity c-reactive protein. ^*^P < 0.05, ^**^P < 0.01, compared with sleep hours ≤7 h. ^†^*P* < 0.05, ^††^*P* < 0.01, compared with sleep hours between 7–9 h.

**Table 3:** Echocardiographic parameters stratified by sleep duration.

	Sleep hours			P_*adjusted1*_	P_*adjusted2*_
	≤7 h	7–9 h	>9 h		
IVSDT, cm	0.92 (0.01)	0.88 (0.01)^**^	0.87 (0.01)^**^	0.002	0.018
LVEDD, cm	4.58 (0.04)	4.40 (0.02)^**^	4.27 (0.04)^**††^	<0.0001	0.003
LVPWT, cm	0.92 (0.01)	0.89 (0.01)^**^	0.87 (0.01)^**^	0.002	0.032
LVM, g	147.2 (3.2)	131.7 (1.8)^**^	121.5 (3.4)^**††^	<0.0001	<0.0001
LVMI, g/m^2.7^	34.68 (0.75)	31.16 (0.42)^**^	29.12 (0.81)^**†^	<0.0001	<0.0001
LVH, n(%)	19 (18.3%)	26 (8%)^*^	16 (6.7%)^**^	0.015	0.087

Data are the means (SE) adjusted for age and sex or N (%). Abbreviations: IVSDT = inter ventricular septal diastolic thickness; LVEDD = left ventricular end-diastolic diameter; LVPWT = left ventricular posterior wall thickness; LVM = left ventricular mass; LVMI = left ventricular mass index; LVH = left ventricular hypertrophy. P_*adjusted1*_ : P value adjusted for age and sex. P_*adjusted2*_: P value adjusted for age, gender, height, weight, waist circumference, blood pressures, lipids, HbA1c, HOMA-IR, leptin, adiponectin, hs-CRP, smoking, physical activity and sleep quality. ^*^P < 0.05, ^**^P < 0.01, compared with sleep hours ≤7 h. ^†^P < 0.05, ^††^*P* < 0.01, compared with sleep hours between 7–9 h.

**Table 4:** Association of sleep duration with cardac-parameters by multivariate regression analysis.

	Model 1		Model 2		Model 3	
	β (SE)	P Value	β (SE)	P Value	β (SE)	P value
IVSDT (cm)	−0.008(0.004)	0.028	−0.008(0.004)	0.018	−0.008(0.004)	0.021
LVEDD (cm)	−0.038(0.013)	0.003	−0.037(0.013)	0.004	−0.036(0.013)	0.005
LVPWT (cm)	−0.009(0.003)	0.004	−0.010(0.003)	0.001	−0.010(0.003)	0.001
LVM (g)	−3.447 (0.912)	<0.0001	−3.533 (0.917)	<0.0001	−3.483 (0.911)	<0.0001
LVMI (g/m^2.7^)	−0.812 (0.209)	<0.0001	−0.824 (0.210)	<0.0001	−0.815 (0.209)	<0.0001

Model 1: adjusted for age, sex, height and weight. Model 2: adjusted for model1 plus triglyceride, HDL-C, blood pressures, HbA1c%, smoking, and physical activity and sleep quality. Model 3: adjusted for model 2 plus hs-CRP, HOMA-IR, adiponectin and leptin. β (SE): parameter estimate from linear regression. Abbreviations: IVSDT = inter ventricular septal diastolic thickness; LVEDD = left ventricular end-diastolic diameter; LVPWT = left ventricular posterior wall thickness; LVM = left ventricular mass; LVMI = left ventricular mass index.
